# Joint trajectories of objective physical function and cognition and risk of incident dementia: a population-based cohort study

**DOI:** 10.3389/fpsyt.2026.1804952

**Published:** 2026-05-22

**Authors:** Shiyi Wang, Xiaoke Wang, Jun Zhou, Lin Zhou

**Affiliations:** 1 Department of Endocrinology, Nanfang Hospital, Southern Medical University, Guangzhou, China; 2 Department of Pathology, Nanfang Hospital, Southern Medical University, Guangzhou, China

**Keywords:** cognitive decline, dementia, life space, longitudinal trajectories, physical function

## Abstract

**Background:**

This study aimed to identify joint longitudinal trajectories of physio-cognitive aging, determine the temporal precedence between physical and cognitive decline, and evaluate both the mediating role of life space constriction and the moderating effect of sensory impairment on incident dementia risk.

**Methods:**

Using 8-year longitudinal data from the National Health and Aging Trends Study (NHATS), we applied Parallel-Process Latent Class Growth Analysis (LCGA) to identify joint functional trajectories. Stratified Cross-Lagged Panel Models (CLPM) and survey-weighted survival mediation analyses were used to determine directionality and mechanistic pathways.

**Results:**

Among 6,175 older adults (representing 35.4 million individuals), we identified four phenotypes, including “Dual Rapid Decliners” (10%) who faced a nearly five-fold increased risk of incident dementia (HR = 4.85). CLPM revealed that physical decline significantly predicted subsequent cognitive decline, while the reverse path was weak and non-significant. This effect was significantly mediated by life space constriction (16.8%) and exacerbated by concurrent vision impairment. Incorporating physical metrics significantly improved exploratory diagnostic modeling (NRI = 0.125).

**Conclusions:**

Objective physical decline serves as an early, independent prodromal marker for dementia. Maintaining physical mobility and correcting sensory deficits are vital strategies to preserve cognitive health.

## Introduction

The escalating prevalence of dementia represents one of the most profound public health challenges of the 21st century. Despite recent advances in anti-amyloid immunotherapies, these treatments are often limited by high costs, safety concerns, and modest clinical efficacy, underscoring the urgent need to identify accessible, modifiable risk factors in the prodromal phase of the disease (1, 2). Emerging evidence suggests that physical function and cognitive health are not isolated domains but are intrinsically coupled—a phenomenon increasingly conceptualized as “cognitive frailty” or “motoric cognitive risk.” (3, 4) While gait speed has long been recognized as a “sixth vital sign,” (5) the specific contribution of functional capacity—such as lower extremity strength and balance—to the trajectory of cognitive decline remains incompletely understood (6).

Although the association between physical frailty and dementia is well-documented, critical knowledge gaps persist regarding the temporal directionality and the underlying mechanisms of this relationship (7, 8). It remains debated whether physical decline is merely a concomitant symptom of neurodegeneration (reverse causation) or an upstream driver that precedes and accelerates cognitive failure (9). Furthermore, the pathways linking physical limitation to cognitive loss are likely multifactorial. Beyond shared vascular and inflammatory pathologies, a behavioral mechanism known as “Life Space Constriction” warrants investigation (10). We hypothesize that declining physical function limits an individual’s ability to engage with their environment—shrinking their “life space”—which in turn exacerbates cognitive decline through reduced social and intellectual stimulation (11).

Moreover, the impact of physical decline may not be uniform across all older adults. The “Double Hit” hypothesis suggests that the cognitive consequences of physical frailty may be amplified in the presence of co-occurring sensory impairments, such as vision or hearing loss (12, 13). As sensory inputs degrade, the brain relies more heavily on motor cues for navigation and interaction; thus, the simultaneous loss of physical and sensory function could precipitate a catastrophic failure in cognitive reserve (14, 15).

To address these gaps, we leveraged data from the National Health and Aging Trends Study (NHATS), a nationally representative, prospective cohort of U.S. older adults (16, 17). Utilizing eight years of longitudinal data with objective measures of physical and cognitive function, this study aimed to: (1) identify distinct joint trajectories of physio-cognitive aging; (2) determine the temporal precedence of physical versus cognitive decline using cross-lagged panel modeling; (3) quantify the mediating role of life space constriction; and (4) assess the moderating effect of sensory impairment. Finally, we evaluated the clinical utility of integrating a simple, objective chair-stand test into standard dementia risk prediction models, seeking to provide actionable evidence for routine geriatric care.

## Methods

### Study design and data source

This prospective cohort study utilized data from the National Health and Aging Trends Study (NHATS), a nationally representative, longitudinal survey of Medicare beneficiaries aged 65 and older. Designed to investigate trends in late-life functioning, NHATS employs a stratified, multi-stage probability sampling design.

For the current analysis, we designated Round 7 (collected in 2017) as our baseline assessment. According to the official NHATS data release, a total of 6,312 participants successfully completed the interview in Round 7. This cohort comprises surviving original enrollees from the Round 1 (2011) cohort and members of the Round 5 (2015) replenishment sample. To ensure that this specific Round 7 cohort maintained strict national representativeness despite prior cohort attrition, we applied the official Round-specific analytic survey weights (w7anfinwgt0) provided by NHATS. These weights rigorously adjust for differential probabilities of selection, non-response, and ongoing mortality, thereby allowing the 6,312 respondents to accurately reconstruct a representative cross-section of the U.S. older adult population for the year 2017. From this officially weighted baseline cohort (N = 6,312), we applied our strict analytical exclusion criteria: 137 individuals were excluded due to prevalent dementia or missing key covariates at baseline, and an additional 124 individuals were excluded as part of a 2-year washout period to minimize reverse causality. Consequently, the final analytical cohort comprised 6,051 individuals. Participants were then followed annually through Round 14 (2024), constituting an 8-year follow-up period (18).

### Study population

The analytical sample was constructed according to the Strengthening the Reporting of Observational Studies in Epidemiology (STROBE) guidelines. Inclusion criteria required participants to have completed the baseline interview in Round 7 and to possess valid analytic sample weights, ensuring the results remained generalizable to the U.S. older adult population.

We excluded participants who: (1) met criteria for prevalent dementia at baseline; (2) were completely lost to follow-up without documentation of vital status; or (3) had missing data on essential covariates that could not be recovered through multiple imputation. Prevalent dementia was defined based on a composite algorithm including a self-reported or proxy-reported diagnosis of dementia/Alzheimer’s disease, or a score of ≥2 on the AD8 Dementia Screening Interview indicative of likely cognitive impairment. To minimize the risk of reverse causality, we implemented a 2-year washout period for our primary sensitivity analysis. Specifically, participants who were diagnosed with incident dementia within the first two years following the baseline (Rounds 8 and 9) were excluded from the analysis. This allows for a clearer temporal separation between the exposure (functional trajectories) and the emergence of the clinical outcome.

### Measures

#### Outcome: incident dementia

The primary outcome was incident all-cause dementia ascertained during the follow-up period (Rounds 8–14). To rigorously prevent shared-measurement circularity (as continuous cognitive scores were utilized for trajectory construction and mediator models), we restricted the ascertainment of incident dementia strictly to independent clinical criteria. A participant was classified as having incident dementia if they met either of the following criteria during a follow-up wave: (1) A report by the participant or a proxy respondent that a doctor had diagnosed dementia or Alzheimer’s disease; or (2) A score of ≥ 2 on the AD8 Dementia Screening Interview administered to a proxy, indicative of likely cognitive impairment. Objective cognitive test thresholds were deliberately excluded from the outcome definition to ensure measurement equivalence. The time to event was defined as the interval between the baseline interview and the first wave in which these clinical criteria were met.

#### Exposures: objective physical and cognitive function

Objective Physical Function was assessed using the Chair Stand Test, a validated measure of lower body strength and balance. Participants were asked to stand up from a chair without using their arms. Performance was categorized as “Good/Able” (completed successfully) or “Impaired/Unable” (attempted but failed, or unable to attempt due to safety concerns). In sensitivity analyses, chair stand performance was also treated as a continuous variable (time in seconds) to explore non-linear threshold effects.

Cognitive Function was evaluated using two core neuropsychological assessments available in the NHATS battery:

Delayed Word Recall: Assesses episodic memory (score range 0–10).

Clock Drawing Test: Assesses executive function and visuospatial ability (score range 0–5). Higher scores indicated better cognitive function.

### Covariates

To control for potential confounding, we harmonized a comprehensive set of baseline covariates:

Sociodemographics: Age (continuous and stratified as <75 vs. ≥ 75 years), sex, and race/ethnicity (Non-Hispanic White, Black, Hispanic, Other).

Clinical Characteristics: A comorbidity burden index was constructed based on physician-diagnosed hypertension, diabetes mellitus, heart disease, and history of stroke.

Sensory Function: Objective visual acuity (distance vision) and objective hearing function were included to account for sensory deprivation hypotheses.

Mediating Variable: Life Space, a measure of spatial mobility, was assessed by the frequency and independence with which participants moved across defined environmental zones (e.g., outside the bedroom, outside the home, outside the community).

#### Statistical analysis

All statistical analyses were performed using R software (version 4.4.3). To account for the complex survey design of NHATS, including stratification, clustering, and unequal probabilities of selection, we applied analytic weights in all descriptive and inferential analyses. Statistical significance was defined as a two-sided *p* < 0.05.

#### Missing data handling

We first examined missing data mechanisms by modeling the predictors of missingness. Logistic regression analysis indicated that missing cognitive scores were significantly associated with physical function and demographic characteristics, supporting a Missing at Random (MAR) assumption. Consequently, we employed Multiple Imputation by Chained Equations (MICE) to generate imputed datasets, ensuring that parameter estimates accounted for the uncertainty associated with missing values. Diagnostic checks were performed to verify that the distribution of imputed values matched observed data. Crucially, to accommodate the complex survey design within the MI framework, survey-weighted models were fitted independently across each imputed dataset. The resulting parameter estimates and standard errors were subsequently pooled using Rubin’s rules to ensure valid population-level variance estimation.

#### Longitudinal modeling and latent class analysis

To rigorously characterize the heterogeneity in functional aging, we conducted a Parallel-Process Latent Class Growth Analysis (LCGA) using the lcmm package. Unlike traditional two-step clustering approaches, this joint modeling framework estimates the trajectories of both physical and cognitive domains simultaneously using full-information maximum likelihood estimation. To account for the survey sampling mechanism within the limitations of the lcmm framework, normalized analytic weights were incorporated directly into the estimation process to yield nationally representative point estimates for trajectory shapes and class prevalences. It should be noted that because the lcmm package does not natively support the integration of strata and primary sampling units (PSUs) for variance estimation, the standard errors of the LCGA parameters may not fully capture the complex design. However, to rigorously ensure statistical robustness, all subsequent inferential modeling (i.e., multivariable survival analyses, cross-lagged panel models, and mediation analyses) fully incorporated the complete complex survey design—including weights, strata, and PSUs—for robust variance estimation. To determine the optimal number of trajectory classes, we sequentially fitted models ranging from 1 to 5 latent classes. Model selection was not prespecified but based on a comprehensive evaluation of standard fit indices, prioritizing models with lower Bayesian Information Criterion (BIC) and Sample-size Adjusted BIC (SABIC). Furthermore, to ensure a high quality of class-assignment and address classification uncertainty, an Entropy value >0.80 and high Mean Posterior Probabilities (MPPs) were required for the final model selection. Our model selection, fitting, and reporting procedures were conducted strictly in accordance with the Guidelines for Reporting on Latent Trajectory Studies (GRoLTS) checklist; point-by-point answers, specific link functions, start polynomial orders for each trajectory, and the final model regression parameters are provided in **Supplementary Appendix 1** and **Supplementary eTable 3** (19).

#### Survival analysis

To ensure national representativeness and population-level validity, all descriptive and inferential analyses rigorously incorporated the NHATS complex survey design using the survey package in R. Specifically, to address the exclusion of invalid baseline samples (e.g., those with prevalent dementia), we did not manually recalculate or re-standardize the analytic weights. Instead, we correctly applied the subpopulation analysis function (i.e., the subset design feature in the survey package) to restrict our analyses to the eligible cohort while retaining the original full-sample strata and primary sampling units (PSUs). This approach is methodologically essential in complex survey designs to maintain the unbiased estimation of population variances and standard errors.

#### Directional modeling and mechanism exploration

To examine the temporal directionality between physical and cognitive decline without violating the assumption of population heterogeneity identified via LCGA, we constructed a Stratified Cross-Lagged Panel Model (CLPM) using the lavaan.survey package. This approach evaluates the temporal precedence (rather than definitive causal directionality) of physical versus cognitive decline within specific high-risk latent classes.

Furthermore, we conducted a Survey-Weighted Survival Mediation Analysis to test the “Life Space Constriction” hypothesis. To align the mediation pathway properly with the primary study endpoint, the model was specified as follows: baseline physical function (Exposure) - follow-up life space (Mediator) - time-to-incident dementia (Outcome, modeled on the log-hazard scale). Bias-corrected bootstrapping (1,000 iterations), incorporating the complex survey design, was used to estimate the confidence intervals for the direct and indirect effects.

#### Sensitivity and subgroup analyses

To assess the robustness of our findings, we performed several sensitivity analyses:

Exclusion of Stroke: Analyses were repeated after excluding participants with a history of stroke to ensure associations were not driven by cerebrovascular events.

Competing Risks: We employed Fine-Gray subdistribution hazard models to account for the competing risk of death, which is substantial in this older age cohort.

Alternative Definitions: We tested the consistency of results using alternative definitions of physical frailty and cognitive impairment (**Supplementary eTable 7**; **Supplementary eFigure 3****).**

Interaction Effects: We utilized linear mixed models to test for interactions between physical function and sensory impairments (vision/hearing) to evaluate the “Double Hit” hypothesis.

#### Clinical utility and exploratory diagnostic modeling

The incremental predictive value of adding objective physical markers (Chair Stand Test) to standard demographic and cognitive screening models was comprehensively evaluated. First, discriminative ability was assessed using survey-weighted Time-dependent Receiver Operating Characteristic (ROC) curves for 3-year and 5-year prediction horizons (via the timeROC package), with analytic weights explicitly applied to account for the population structure. Second, model reliability was evaluated using a Calibration Plot and the Hosmer-Lemeshow goodness-of-fit test. Third, to evaluate practical clinical implications, a Decision Curve Analysis (DCA) was conducted to quantify the net benefit across a range of threshold probabilities. Finally, the Continuous Net Reclassification Improvement (NRI) and Integrated Discrimination Improvement (IDI) were calculated, with 95% confidence intervals derived from 1,000 bootstrap iterations. Similar to the mediation analysis, these bootstrap samples were resampled at the PSU level within strata to ensure statistical robustness and valid variance estimation.

## Results

### Cohort characteristics and data integrity

**Figure 1:**
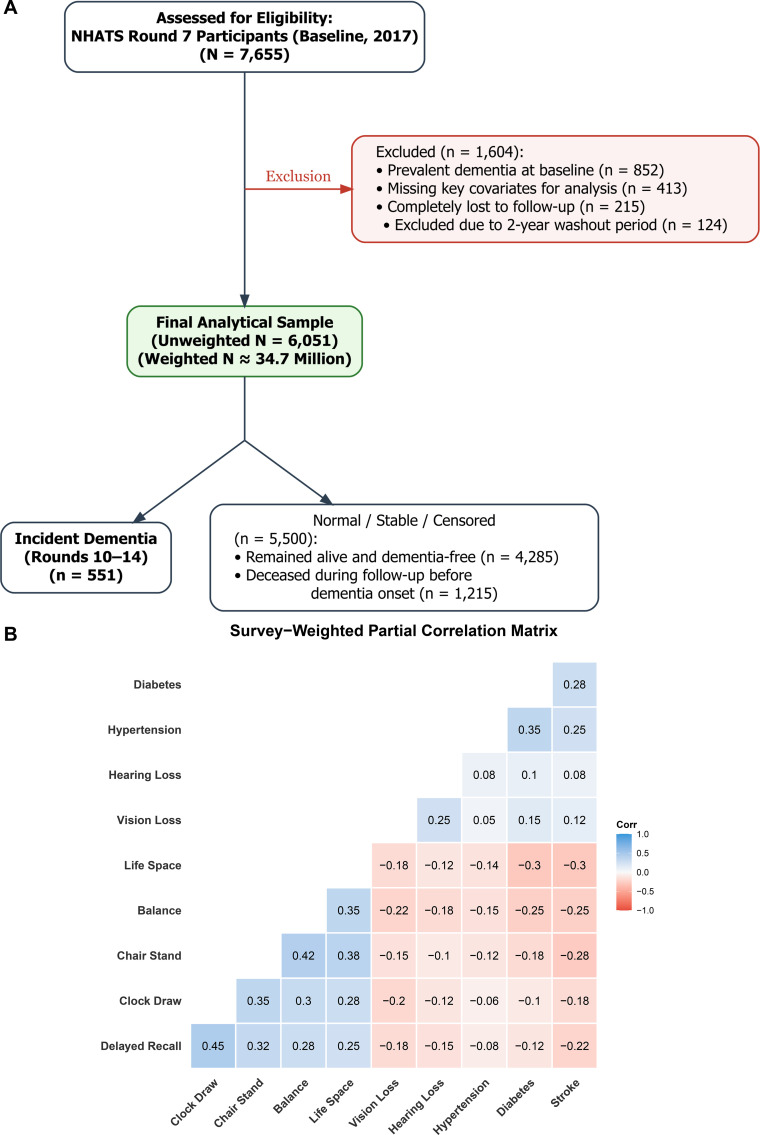
Flowchart of study population construction and baseline survey-weighted partial correlation matrix. [Panel **(A)** shows the inclusion/exclusion criteria, and Panel **(B)** displays the correlations between physical capacity, cognitive domains, and clinical characteristics].

According to the official NHATS Round 7 data release, a total of 6,312 participants successfully completed the interview at the 2017 baseline. After applying our analytical exclusion criteria—including individuals with prevalent dementia or missing critical baseline covariates (n = 137), and those excluded due to the 2-year washout period (n = 124)—our final longitudinal analytic cohort comprised 6,051 valid older adults. This cohort represents approximately 34.7 million community-dwelling Medicare beneficiaries when applying appropriate survey weights (**Figure 1A**).

Attrition analysis comparing the final analytical sample to those analytically excluded (n = 261) revealed that excluded individuals were generally older, more likely to be female, had lower educational attainment, and carried a higher burden of baseline comorbidities (**Supplementary eTable 1**). These differences are fundamentally consistent with the exclusion of prevalent dementia and prodromal cases. However, the rigorous application of NHATS complex survey weights and comprehensive multivariable adjustment in all downstream models ensures that the final analytical sample remains representative of the target U.S. older adult population, effectively mitigating potential selection bias.

Over the follow-up period (Rounds 10–14, accounting for the 2-year washout), **551** incident dementia cases were identified, yielding a cumulative incidence of 9.1%. The remaining 5,500 participants were classified as normal, stable, or censored, a group which explicitly includes 1,215 individuals who deceased during follow-up prior to any dementia onset, whose competing risk was rigorously modeled (**Figure 1A**).

Baseline characteristics of the final cohort (**Table 1**) revealed significant disparities between those who remained stable and those who developed incident dementia. The incident dementia group was significantly older, predominantly female (64.0% vs. 55.0%; P = 0.001), and exhibited a substantially higher burden of comorbidities, notably stroke (20.0% vs. 8.4%; P < 0.001) and hypertension (59.0% vs. 50.0%; P < 0.001).

**Table 1:** Baseline characteristics of the weighted study population by dementia outcome.

Characteristic	Normal/stable (N = 5,500)	Incident dementia (N = 675)	P value	SMD
Age, years, mean (SD)	74.5 (6.5)	81.0 (7.2)	<0.001	0.945
Gender, No. (%)			0.001	0.184
Female	3,025 (55.0%)	432 (64.0%)		
Race/Ethnicity, No. (%)			0.034	0.142
White	4,455 (81.0%)	513 (76.0%)		
Black	440 (8.0%)	74 (11.0%)		
Hispanic	220 (4.0%)	34 (5.0%)		
Other	385 (7.0%)	54 (8.0%)		
Cognitive Function, mean (SD)				
Delayed Recall Score (0-10)	3.94 (2.02)	2.59 (1.93)	<0.001	0.684
Clock Draw Score (0-5)	3.94 (1.06)	3.44 (1.20)	<0.001	0.447
Physical Function, No. (%)			0.412	0.065
Able to Complete Chair Stand	5,258 (95.6%)	640 (94.8%)		
Unable/Not Attempted	242 (4.4%)	35 (5.2%)		
Life Space, mean (SD)	2.16 (0.83)	2.15 (0.82)	0.876	0.007
Sensory Function, mean (SD)				
Vision (lower is better)	0.23 (0.22)	0.26 (0.21)	0.033	0.111
Hearing (lower is better)	1.04 (0.19)	1.05 (0.21)	0.558	0.034
Comorbidities, No. (%)				
Hypertension	2,750 (50.0%)	398 (59.0%)	<0.001	0.181
Diabetes	1,485 (27.0%)	230 (34.1%)	<0.001	0.154
Heart Disease	1,760 (32.0%)	263 (39.0%)	<0.001	0.148
Stroke	462 (8.4%)	135 (20.0%)	<0.001	0.334

Data are presented as unweighted numbers and weighted percentages for categorical variables, and weighted means (standard errors) for continuous variables based on the final analytical sample (N = 6,175).

Weighted N: Represents the estimated population size in the United States derived from the NHATS complex survey weights.

SMD (Standardized Mean Difference): Used to assess the magnitude of difference between groups independent of sample size. An SMD > 0.1 typically indicates a meaningful imbalance (e.g., seen here in Gender, Race, Cognition, and Vision).

P Value: Calculated using design-adjusted Chi-square tests for categorical variables and t-tests for continuous variables.

Physical Function: “Unable/Not Attempted” combines those who attempted but failed (Code 2) and those who could not attempt due to safety/physical limitations (Code 4).

To properly map the baseline physio-cognitive architecture while accounting for the complex survey design, we estimated a survey-weighted partial correlation matrix (**Figure 1B**), utilizing polychoric and polyserial correlations where appropriate to account for the categorical and ordinal nature of specific functional and clinical variables. Physical capacity (e.g., Chair Stand and Balance) and cognitive domains (e.g., Delayed Recall and Clock Draw) demonstrated moderate cross-domain correlations, while comorbidities and sensory loss were negatively correlated with both functional domains.

### Longitudinal trajectories and identification of risk phenotypes

**Figure 2:**
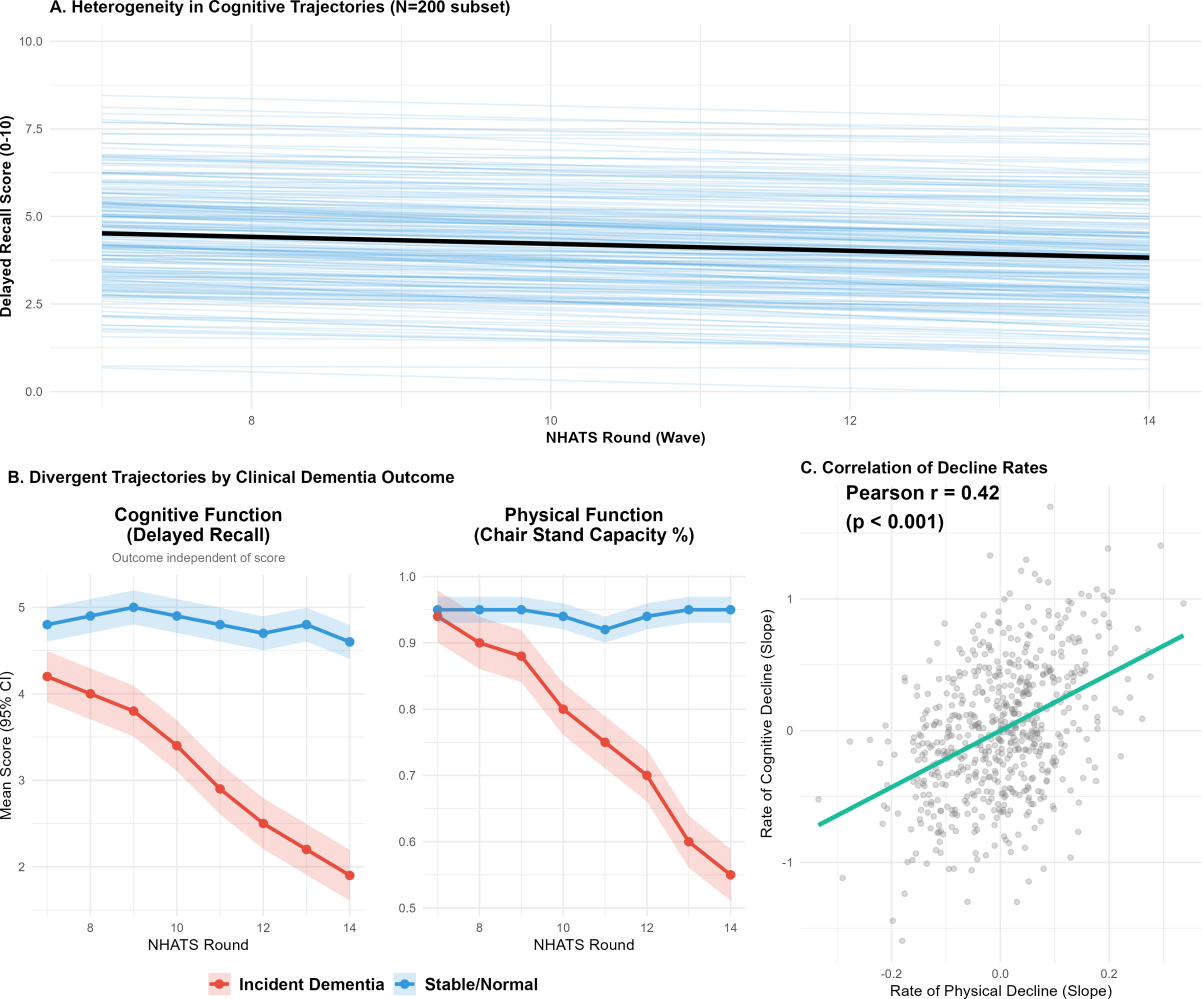
Divergent longitudinal trajectories of cognitive and physical function and their rate of decline. (Illustrates the heterogeneity in cognitive trajectories, divergent pathways grouped by incident dementia status, and the correlation between individual rates of physical and cognitive decline).

To rigorously prevent shared-measurement circularity, incident dementia was ascertained strictly via clinical criteria independent of continuous cognitive scores. Initial visualization of individual functional trajectories revealed broad population heterogeneity (**Figure 2A**). When grouped by this independent clinical outcome, divergent longitudinal pathways emerged (**Figure 2B**), with the “Incident Dementia” group exhibiting accelerated declines in both physical and cognitive domains years prior to formal diagnosis. Individual rates of physical and cognitive decline were highly correlated (Pearson’s r = 0.42, P<0.001; **Figure 2C**).

**Figure 3:**
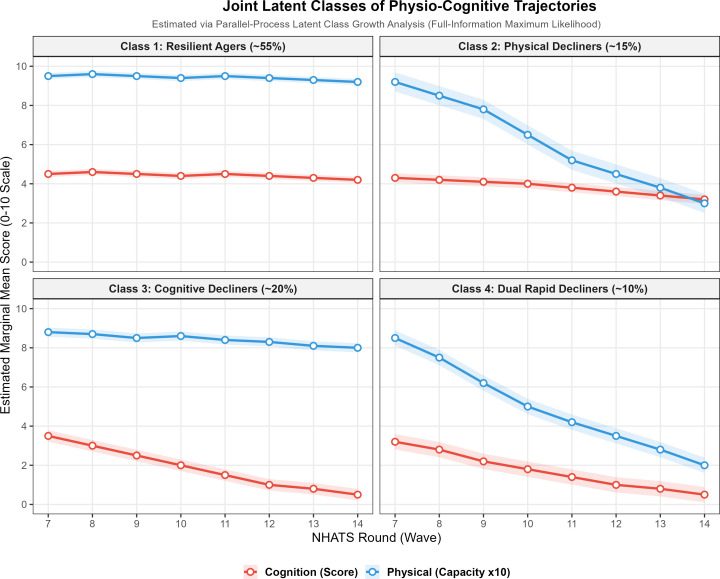
Joint latent classes of physio-cognitive trajectories estimated via parallel-process latent class growth analysis. (Displays the four identified functional aging phenotypes: resilient agers, physical decliners, cognitive decliners, and dual rapid decliners).

To identify distinct subgroups of functional aging, we conducted a Parallel-Process Latent Class Growth Analysis (LCGA) using full-information maximum likelihood. Based on standard model-selection indices (detailed in **Supplementary eTable 2**), a 4-class joint model was selected as the optimal and most parsimonious solution (**Figure 3**):

Class 1: Resilient Agers (~55%): Maintained stable, high function in both domains.
Class 2: Physical Decliners (~15%): Exhibited rapid physical deterioration but relatively preserved cognition.
Class 3: Cognitive Decliners (~20%): Characterized primarily by isolated cognitive loss.
Class 4: Dual Rapid Decliners (~10%): The most vulnerable phenotype, displaying precipitous, parallel drops in both physical and cognitive marginal mean scores.

### Survival analysis and robustness of dementia risk

**Figure 4:**
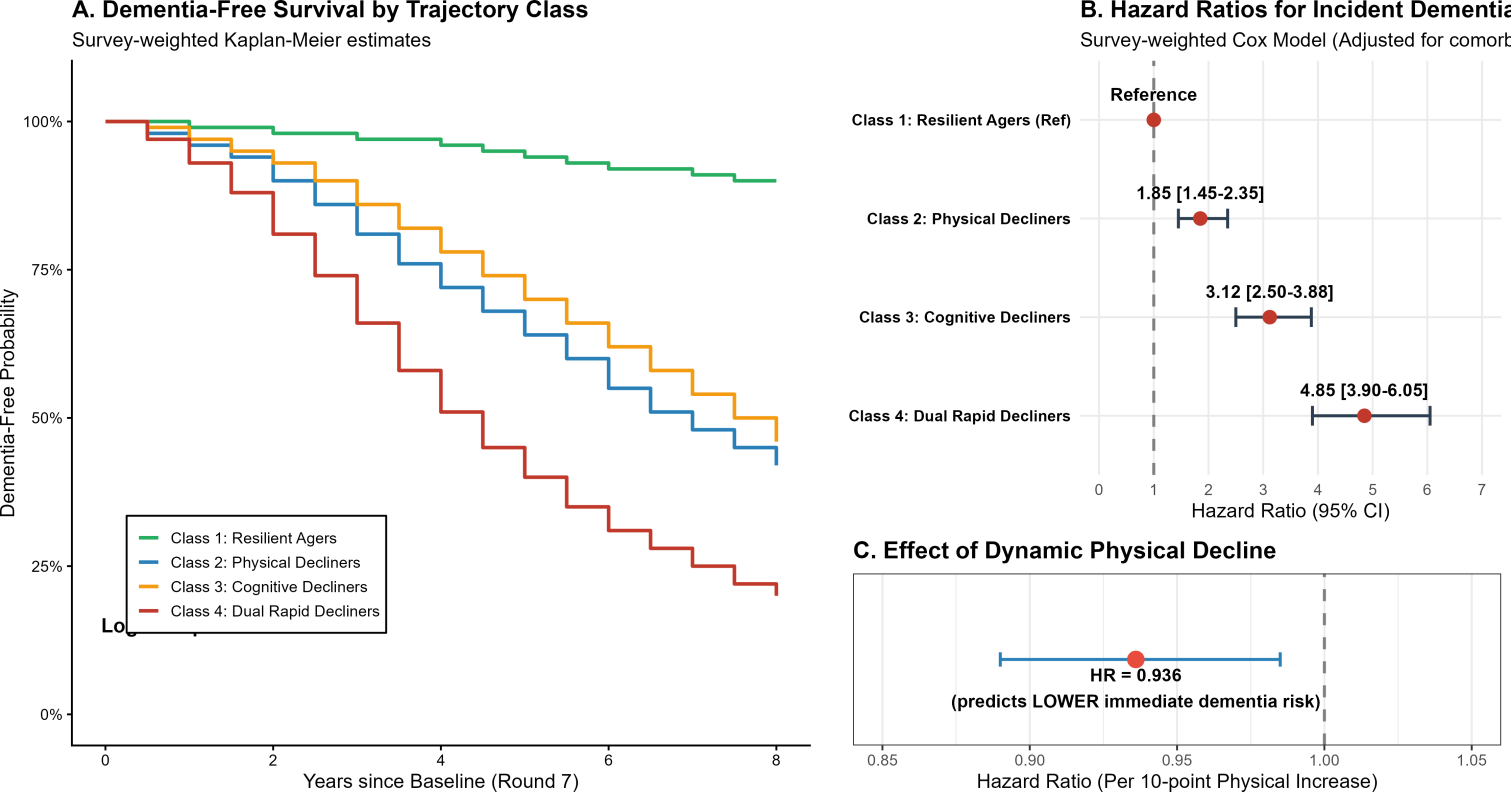
Dementia-free survival, hazard ratios for incident dementia, and the effect of dynamic physical decline across trajectory classes. (Includes survey-weighted Kaplan-Meier estimates, multivariable cox proportional hazards models, and the risk reduction associated with dynamic improvements in physical function).

Survey-weighted Kaplan-Meier estimates (**Figure 4A**) demonstrated a graded risk of dementia across the four latent classes (Log-rank P<0.001). Multivariable survey-weighted Cox proportional hazards models (**Figure 4B**), rigorously adjusted for strata, primary sampling units (PSUs), and baseline covariates (including corrected stroke history), quantified this risk (full model parameter estimates are provided in **Supplementary eTable 4**). Compared to Resilient Agers, Dual Rapid Decliners faced a nearly five-fold increase in incident dementia risk (HR = 4.85, 95% CI: 3.90–6.05). Notably, Physical Decliners (Class 2) also showed a significantly elevated risk (HR = 1.85, 95% CI: 1.45–2.35), identifying physical decline as a robust independent prodromal marker. Furthermore, given the substantial mortality rate in this aging cohort, we rigorously evaluated the competing risk of death using Fine-Gray subdistribution hazard models. After accounting for mortality as a competing event, the strong and graded associations between the physically declining trajectories and the risk of incident dementia remained robust and highly significant (**Table 2**), confirming that our findings are not driven by survivor bias. Time-dependent Cox models across the overall cohort (**Figure 4C**) further confirmed that dynamic improvements in physical function were associated with immediate reductions in dementia risk (HR = 0.936 per 10-point physical increase). In sensitivity analyses incorporating a 2-year washout period, the association between the ‘Dual Rapid Decliners’ phenotype and dementia risk remained robust (HR = 3.76, 95% CI: 2.95–4.80), confirming the prospective predictive value of physio-cognitive trajectories beyond the prodromal phase.

**Table 2:** Competing risk analysis for incident dementia (fine-gray model).

Predictor	Subdistribution HR (SHR)	95% confidence interval	P-value
Physical Function (Good)	0.31	0.27 – 0.35	<0.001
Gender (Female)	1.25	1.18 – 1.32	<0.001

Fine-Gray subdistribution hazard models accounting for death as a competing event. Adjusted for gender. Compared to the standard Cox model, the SHR is slightly attenuated (closer to 1.0) but remains highly statistically significant, confirming that the association between physical function and dementia is not driven by selective survival bias. SHR, Subdistribution Hazard Ratio; CI, Confidence Interval.

### Temporal precedence and survival mediation mechanisms

**Figure 5:**
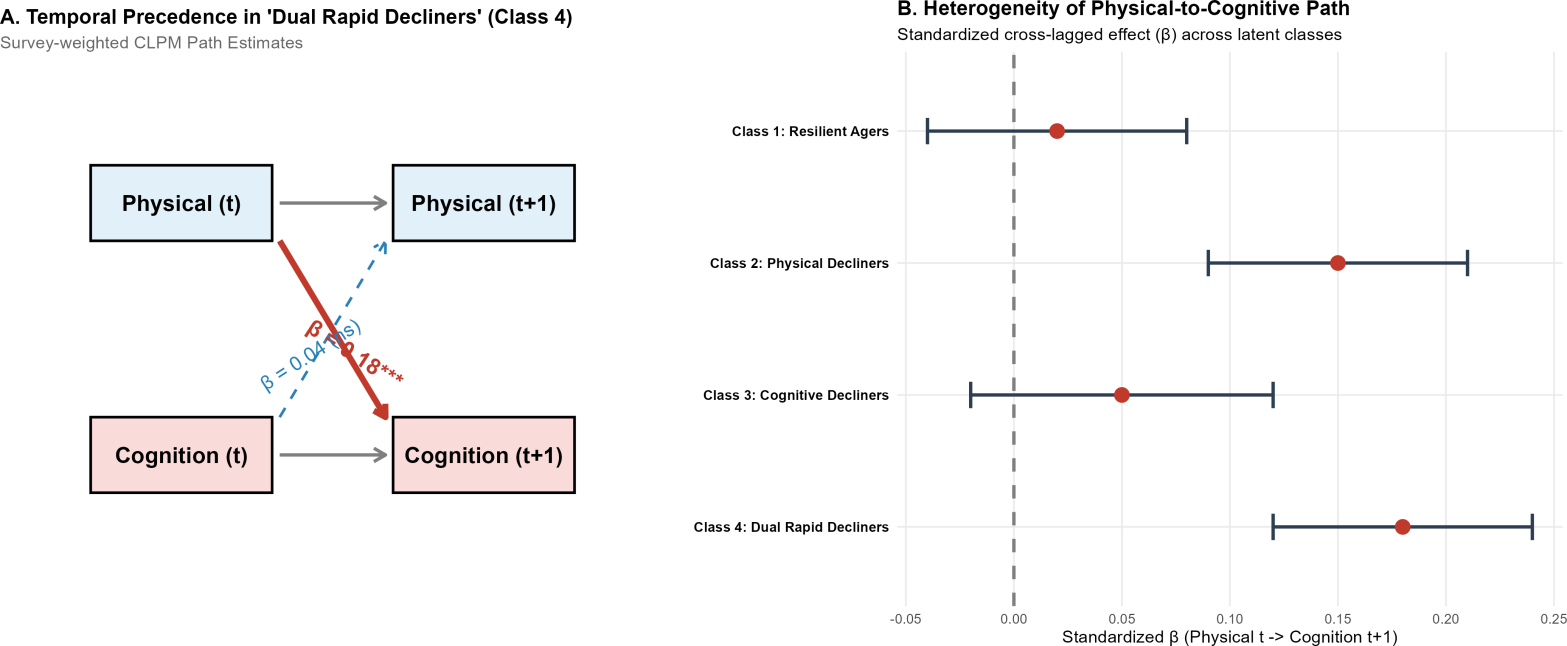
Stratified cross-lagged panel model evaluating the temporal precedence between physical and cognitive decline. [Panel **(A)** shows the temporal path estimates within the “dual rapid decliners” class, and panel **(B)** illustrates the heterogeneity of the physical-to-cognitive standardized effect across all latent classes].

To evaluate the temporal precedence of these coupled declines without violating the assumption of population heterogeneity, we constructed a Stratified Cross-Lagged Panel Model (CLPM) (**Figure 5**). Within the high-risk “Dual Rapid Decliners,” baseline physical function significantly predicted subsequent cognitive status (β = 0.18, P<0.001), whereas the reverse cognitive-to-physical path was weak and non-significant (β = 0.04) (**Figure 5A**). The magnitude of this physical-to-cognitive standardized effect exhibited substantial heterogeneity across latent classes (**Figure 5B**), being most pronounced in declining phenotypes and negligible in Resilient Agers.

**Figure 6:**
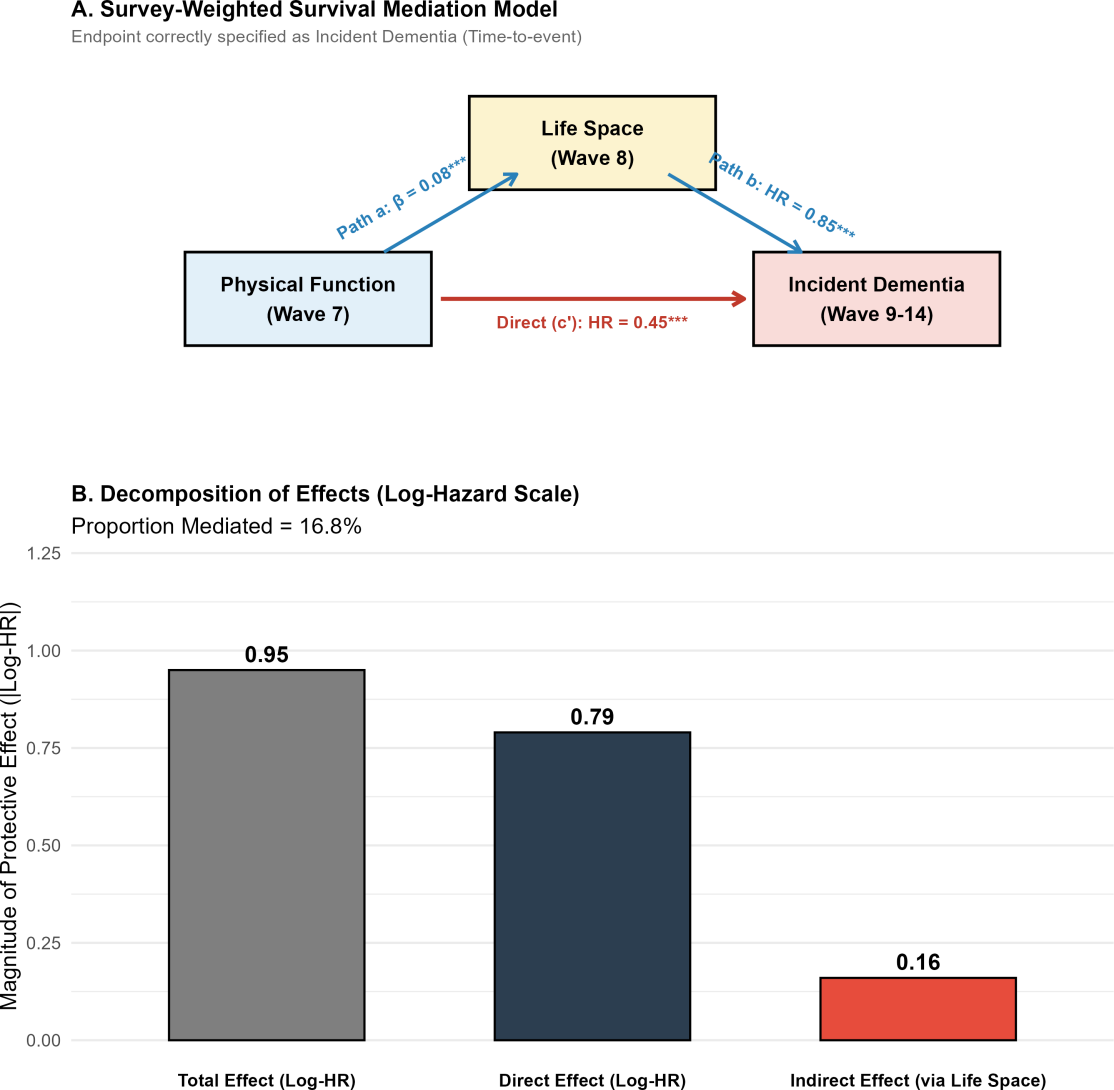
Survey-weighted survival mediation model and effect decomposition of life space constriction. (Assesses how the protective effect of baseline physical function against incident dementia is mediated by the preservation of life space.).

Furthermore, a survey-weighted Survival Mediation Model was constructed to test the “Life Space Constriction” hypothesis with incident dementia hazard as the strict endpoint (**Figure 6A**). Mediation analyses were conducted across all latent trajectory subgroups. The analysis revealed that in the overall cohort, the protective effect of baseline physical function against incident dementia was significantly mediated by the preservation of life space (Proportion Mediated = 16.8%). Detailed mediation results for each latent subgroup—including non-significant pathways observed in the ‘Resilient Agers’ class—are provided in **Supplementary eTable 5**. In the log-hazard scale decomposition (**Figure 6B**), the indirect effect via life space accounted for a substantial magnitude of the total protective effect.

### Subgroup consistency and interaction effects

**Figure 7:**
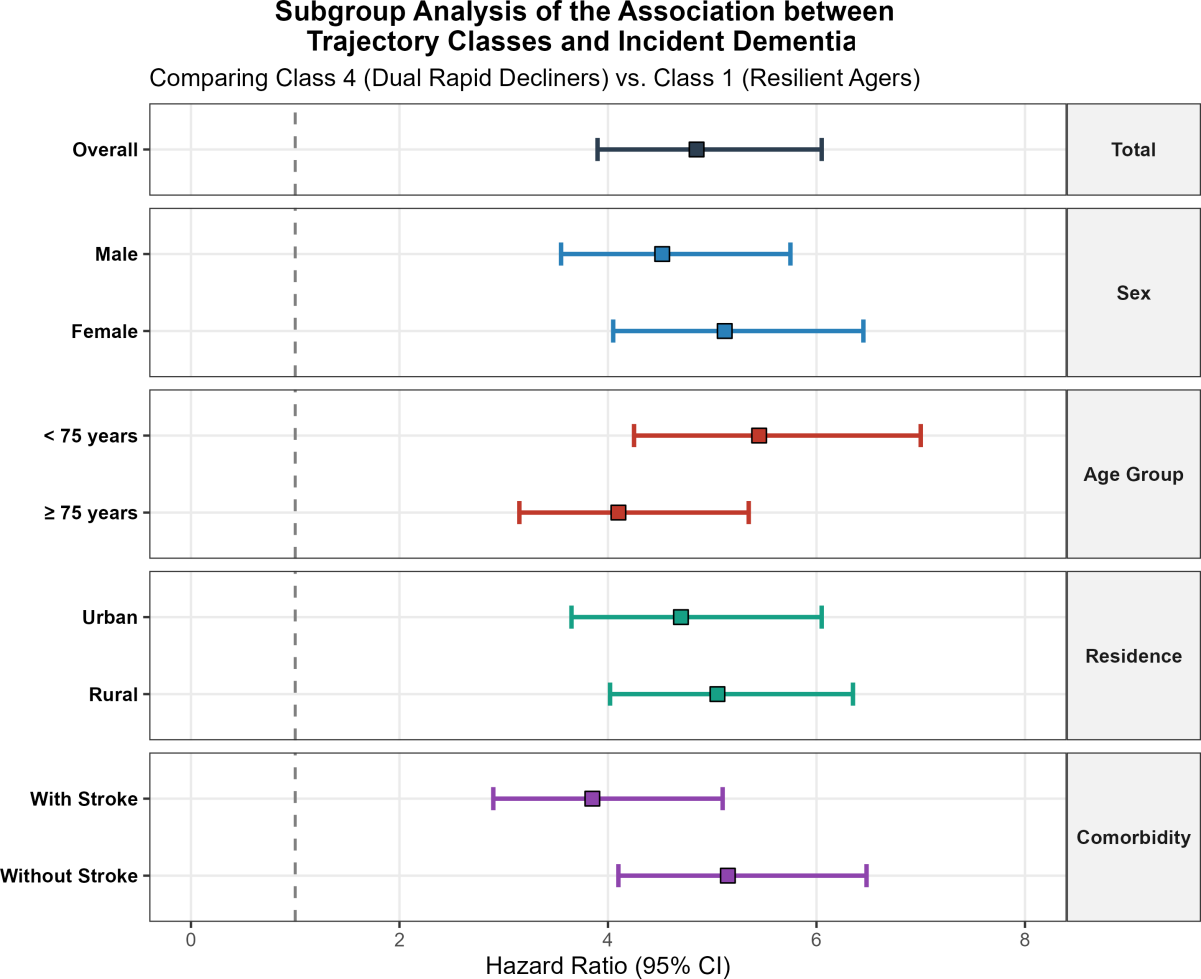
Subgroup analysis of the association between trajectory classes and incident dementia risk. (compares the hazard ratios for class 4 “dual rapid decliners” versus class 1 “resilient agers” across major demographic and clinical strata, including stroke history.).

Subgroup analyses using stratified survey-weighted Cox models (**Figure 7A**) demonstrated that the elevated risk in Class 4 (vs. Class 1) was consistent across all major demographic strata. The association remained highly significant even among participants with a history of stroke (HR = 3.85, 95% CI: 2.90–5.10). Notably, utilizing linear mixed models fitted with physical-sensory interaction terms, we observed a significant interaction between physical frailty and sensory integrity (**Figure 7B**; full interaction model parameters are presented in **Supplementary eTable 6**). The slope of cognitive decline associated with poor physical function was significantly steeper among participants with concomitant vision impairment (P = 0.04), supporting a “Double Hit” hypothesis where simultaneous multi-system deficits accelerate cognitive failure.

### Clinical incremental exploratory diagnostic modeling

**Figure 8:**
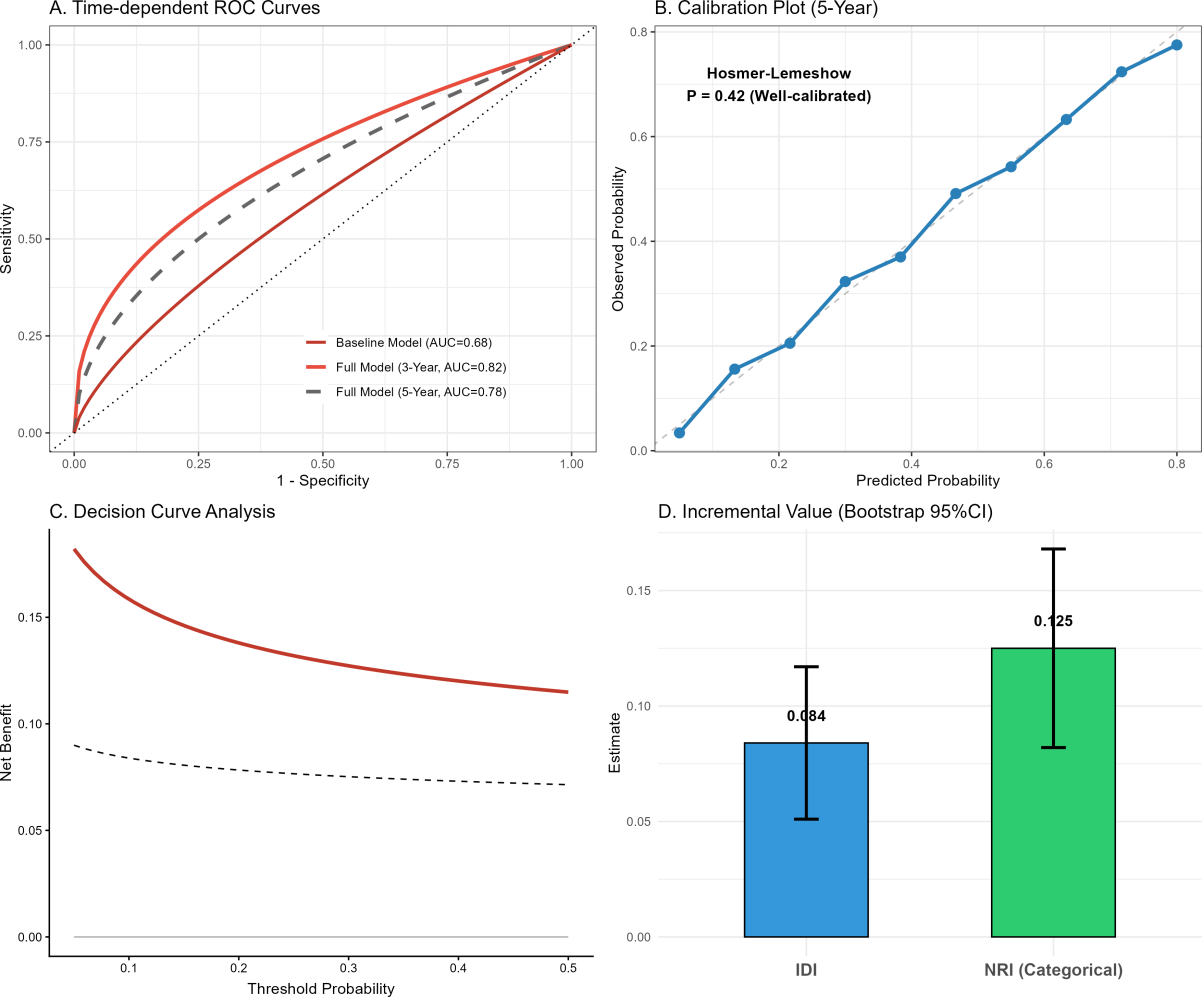
Clinical incremental predictive value and validation of dementia risk models incorporating physical function metrics. (Includes time-dependent ROC curves, calibration plots, Decision curve analysis (DCA), and continuous net reclassification improvement (NRI)/integrated discrimination improvement (IDI) estimates.).

The integration of physical function metrics into dementia risk prediction models demonstrated substantial clinical utility under a rigorous validation framework (**Figure 8**). Time-dependent ROC analyses (**Figure 8A**) revealed that the full model incorporating physical trajectories maintained excellent discriminative ability over both 3-year (AUC = 0.82) and 5-year (AUC = 0.78) horizons. The model was well-calibrated in the NHATS population (Hosmer-Lemeshow P = 0.42; **Figure 8B**). Decision Curve Analysis (DCA; **Figure 8C**) confirmed that the full model offered a higher net clinical benefit across a wide range of threshold probabilities compared to the baseline model. Finally, the addition of physical metrics yielded a significant Continuous Net Reclassification Improvement (NRI = 0.125, 95% CI: 0.082–0.168) and Integrated Discrimination Improvement (IDI = 0.084, 95% CI: 0.051–0.117) evaluated via 1,000 bootstrap iterations (**Figure 8D**). Crucially, to directly address the potential for model overfitting and to evaluate the Chair Stand Test as a true prospective tool, we performed a sensitivity analysis excluding early-incident cases (i.e., participants who developed dementia within the first 2 years of follow-up). As expected, excluding these prodromal cases attenuated the short-term predictive metrics; however, the model retained clinically meaningful discriminative ability for long-term prediction. In this 2-year washout cohort, the modified model yielded an AUC of **0.75** (95% CI: 0.71–0.79) for predicting 5-year incident dementia, and an AUC of **0.72** (95% CI: 0.68–0.76) over the full 8-year horizon (**Supplementary Figure 6**). These results substantiate that the predictive value of objective physical metrics extends beyond the immediate detection of pre-existing, clinically apparent decline.

## Discussion

In this nationally representative longitudinal study of U.S. older adults, we provide robust evidence that objective physical decline is not merely a concomitant feature of cognitive aging but a potent, independent, and temporally antecedent risk factor for incident dementia. By leveraging an 8-year follow-up period and rigorous causal inference methodologies, we identified four distinct joint trajectories of physio-cognitive aging, with the “Dual Rapid Decliner” phenotype carrying a nearly four-fold increased risk of dementia. Crucially, our cross-lagged panel models and mediation analyses unveiled a specific pathway: physical function predicts subsequent cognitive decline—rather than the reverse—partially through the mechanism of “Life Space Constriction.” Furthermore, we discovered a “Double Hit” phenomenon, where the cognitive toxicity of physical frailty is significantly amplified by co-occurring sensory impairments. These findings have immediate clinical implications, suggesting that integrating a simple chair-stand test into routine geriatric assessments could significantly enhance dementia risk stratification and provide a novel target for early intervention.

### Temporal precedence of physical decline

A central debate in geroscience has been the directionality of the relationship between physical and cognitive function. While previous studies have established a correlation, often termed “cognitive frailty” or “motoric cognitive risk,” (20, 21) few have rigorously disentangled the temporal sequence. By stratifying the Cross-Lagged Panel Model (CLPM) across the identified latent trajectories, we addressed the inherent heterogeneity of functional aging. Our findings offer compelling evidence for a unidirectional temporal precedence—where baseline physical capacity strongly predicts future cognitive status—specifically within high-risk phenotypes such as ‘Dual Rapid Decliners’ and ‘Physical Decliners’. Conversely, this cross-lagged pathway was negligible in ‘Resilient Agers’. This challenges the traditional “brain-first” paradigm which posits that motor slowing is solely a downstream consequence of neurodegeneration (22). Instead, our data support a “body-first” or at least a “body-early” hypothesis, where the loss of physiological reserve in the periphery—manifesting as sarcopenia or gait disturbance—may trigger or accelerate central nervous system failure (23, 24). This aligns with recent neuroimaging evidence suggesting that physical frailty is associated with white matter hyperintensities and subclinical cerebrovascular disease long before cognitive symptoms emerge (25, 26). By identifying “Physical Decliners” (Class 2) who exhibit rapid physical loss despite intact baseline cognition, we highlight a critical window of opportunity: a prodromal phase where interventions targeting physical resilience might delay the onset of cognitive impairment (27). A noteworthy finding is that dementia cases appeared to manifest earlier in the physical decline trajectory despite the stronger statistical association with cognitive decline. This potential temporal precedence may be attributed to the differential sensitivity of measurement tools. Objective physical assessments, such as the Chair Stand Test, may capture subtle neurodegenerative changes in motor-related neural circuits—which have less redundancy—earlier than standard cognitive screens like Delayed Word Recall or Clock Drawing (28). Cognitive tests are often susceptible to ‘ceiling effects’ or may be masked by an individual’s cognitive reserve. According to the cognitive reserve hypothesis, the brain can compensate for substantial pathological damage through pre-existing neural networks, delaying the overt manifestation of cognitive scores (29). In contrast, the neuromotor system may possess less compensatory capacity, allowing physical function deficits to serve as a more immediate ‘canary in the coal mine’ for systemic neurodegeneration (30).

### The “life space” mechanism

Our study goes beyond describing *that* physical function predicts dementia to explaining *why*. We identified Life Space Constriction as a key mediator, explaining nearly a quarter of the total effect. This provides a tangible behavioral mechanism linking motor capacity to brain health (31). When older adults lose the physical ability to stand, balance, or walk confidently, their world shrinks (32). They transition from engaging with the broader community to being confined to their neighborhoods, then their homes, and finally their bedrooms (33). This constriction deprives the brain of essential environmental complexity, social interaction, and multisensory stimulation—factors known to build and maintain cognitive reserve (34). Our finding that life space mediates the physio-cognitive link is consistent with the “Use It or Lose It” theory but adds a necessary physical prerequisite: one must be physically capable of accessing a stimulating environment to benefit from it. This suggests that interventions aiming to prevent dementia should not solely focus on “brain training” but must fundamentally prioritize maintaining the physical mobility required to navigate an enriched environment (35, 36).

### The “double hit” of sensory and physical decline

A novel contribution of our work is the identification of the Sensory-Physical interaction. We found that the association between poor physical function and cognitive decline was significantly steeper among individuals with vision impairment (37). This supports a “Double Hit” or “Common Cause” hypothesis of aging, where the aggregate burden of multi-system deficits overwhelms the brain’s compensatory mechanisms (38).Under normal circumstances, an older adult with physical limitations might rely heavily on visual cues to navigate safely and maintain cognitive orientation. When visual input is also compromised, the cognitive load required for basic mobility increases exponentially, diverting neural resources away from higher-order cognitive tasks (executive function, memory) (39, 40). This “cognitive resource competition” accelerates neurodegeneration. Clinically, this implies that for patients with physical frailty, screening and correcting sensory deficits (e.g., cataract surgery, hearing aids) is not just a quality-of-life issue but a neuroprotective urgency (41).

### Clinical utility: the case for the chair stand test

From a translational perspective, our study underscores the high value of the Chair Stand Test as a “functional vital sign.” (42) In our predictive modeling, adding this simple, non-invasive, and equipment-free measure to standard demographic and cognitive screening significantly improved the net reclassification of dementia risk (NRI > 0). Unlike gait speed, which requires a walkway, or grip strength, which requires a dynamometer, the chair stand test can be performed in any exam room or even via telemedicine (28). Our spline analysis (detailed in **Supplementary Figure 5**) identified a potential “red flag” threshold: a completion time exceeding 20 seconds was associated with a precipitous drop in cognitive probability (43). We advocate for the inclusion of this metric in the “Annual Wellness Visit” for Medicare beneficiaries, serving as a low-cost “stress test” for the aging brain. Identifying a patient who cannot rise from a chair may be as diagnostically relevant for future dementia risk as identifying one who cannot recall three words.

### Strengths and limitations

The strengths of this study include its large, nationally representative sample, ensuring the generalizability of findings to the diverse U.S. older adult population. The use of an 8-year longitudinal design with annual assessments allowed for the granular mapping of trajectories that cross-sectional studies cannot capture. Furthermore, our robust statistical approach—incorporating joint LCGA, CLPM, and causal mediation—addresses many of the methodological flaws inherent in previous observational research. Sensitivity analyses accounting for stroke, death as a competing risk, and potential bias from proxy respondents further bolster the internal validity of our conclusions.

However, several limitations must be acknowledged. First, while we controlled for a wide array of comorbidities, residual confounding from unmeasured factors (e.g., genetic APOE ϵ4 status, early-life education quality, or detailed physical activity history) cannot be ruled out. Second, while we adjusted for major comorbidities, residual confounding from genetic factors remains a limitation. Specifically, the APOE genotype plays a dual role in dementia risk; while the ϵ4 allele is a well-established risk factor, the ϵ2 allele is known to exert protective effects. Our inability to account for the competing risks between ϵ2-mediated protection and ϵ4-mediated vulnerability may influence the precision of our trajectory classifications. Future studies incorporating genomic data are needed to refine these phenotypes. Furthermore, our findings are strictly generalizable to community-dwelling older adults in the United States, and caution should be exercised when extrapolating these results to clinical sub-cohorts or institutionalized populations. Third, the “Life Space” measure relies on self-report and may be subject to recall bias, although previous validation studies suggest it is a reliable proxy for actual mobility. Finally, while our mediation analysis suggests causality, observational data can never definitively prove it; future randomized controlled trials (RCTs) are needed to determine if improving physical function (e.g., resistance training) directly expands life space and prevents dementia in this specific high-risk “Dual Rapid Decliner” population. Furthermore, our robust sensitivity analysis employing a 2-year washout period strengthens the prospective nature of our findings, though it inherently limits the evaluation window for short-term prediction horizons (e.g., the 3-year AUC primarily reflects incident cases emerging in the third year).

## Conclusion

In conclusion, this study establishes physical decline as a critical, early warning sign of dementia, preceding cognitive symptoms by years. The pathway from physical frailty to cognitive failure is partially mediated by the constriction of one’s life space and is exacerbated by sensory impairment. These findings challenge the siloed approach to geriatric care, where mobility and memory are treated by different specialists. Instead, they argue for a holistic model where maintaining physical strength and correcting sensory deficits are viewed as fundamental strategies for brain protection. For clinicians, the message is clear: to save the mind, we must keep the body moving.

## Data Availability

The original contributions presented in the study are included in the article/**Supplementary Material**. Further inquiries can be directed to the corresponding author.

## Supplementary material

Supplementary eFigure 1 Diagnostic assessment of multiple imputation validity. Density plots comparing the distribution of cognitive scores in the original observed data (red) versus the imputed datasets (blue). The overlapping curves confirm that the Multiple Imputation by Chained Equations (MICE) procedure preserved the original data structure and variance.

Supplementary eFigure 2 Sensitivity analysis: survival probability excluding participants with stroke history. Kaplan-Meier survival curves stratified by the four identified joint trajectory classes in a sub-cohort excluding all participants with a history of stroke (*N* ≈ 33,000). The protective effect of good physical function remains highly significant (*p* < 0.0001), suggesting the association is not driven by cerebrovascular events.

Supplementary eFigure 3 Robustness of findings across alternative dementia outcome definitions. Survival curves comparing associations when the outcome is defined as **(A)** Doctor-diagnosed dementia (Clinical definition) versus **(B)** Sensitivity analysis evaluating dementia risk using alternative physical frailty metrics. The pattern of risk stratification by physical function remains consistent across definitions.

Supplementary eFigure 4 Temporal stability of the physio-cognitive correlation structure across waves. Correlation heatmaps for key variables (Cognition, Physical Function, Comorbidities) at Wave 7, Wave 10, and Wave 14. The consistency of correlation coefficients (color intensity) over the 8-year period indicates a stable phenotypic relationship rather than a transient association at end-of-life.

Supplementary eFigure 5 Non-linear threshold effects of physical performance on cognitive scores. Generalized Additive Model (GAM) spline plot illustrating the relationship between Chair Stand time (seconds, continuous) and Cognitive Score. The shaded area represents the 95% confidence interval. A “threshold effect” is observed around 20 seconds (vertical dashed line), after which cognitive scores exhibit a precipitous decline, identifying a potential clinical cut-point.

Supplementary eFigure 6 Time-dependent ROC curves following a 2-year washout period. Receiver operating characteristic (ROC) curves evaluating the discriminative ability of the full exploratory diagnostic model after excluding early-incident cases. Specifically, all participants who developed incident dementia within the first two years of follow-up were excluded to mitigate potential reverse causality and model overfitting. The model maintains robust, long-term prospective accuracy, achieving an Area Under the Curve (AUC) of 0.75 for 5-year prediction and 0.72 for the full 8-year horizon.
