# Correction: Joint trajectories of objective physical function and cognition and risk of incident dementia: a population-based cohort study

**DOI:** 10.3389/fpsyt.2026.1918426

**Published:** 2026-07-08

**Authors:** Shiyi Wang, Xiaoke Wang, Jun Zhou, Lin Zhou

**Affiliations:** 1 Department of Endocrinology, Nanfang Hospital, Southern Medical University, Guangzhou, China; 2 Department of Pathology, Nanfang Hospital, Southern Medical University, Guangzhou, China

**Keywords:** cognitive decline, dementia, life space, longitudinal trajectories, physical function

Affiliation “Department of Pathology, Nanfang Hospital, Southern Medical University, Guangzhou, China” was omitted for authors Xiaoke Wang and Jun Zhou. This affiliation has now been added for authors Xiaoke Wang and Jun Zhou.

The original version of this article has been updated.

