# Supplementary material for: Joint trajectories of objective physical function and cognition and risk of incident dementia: a population-based cohort study
Source: Front Psychiatry. 2026 May 22;17:1804952. doi: 10.3389/fpsyt.2026.1804952 (PMC13236857; doi:10.3389/fpsyt.2026.1804952)
Supplement: Supplementary eFigure 1 — Diagnostic assessment of multiple imputation validity. Density plots comparing the distribution of cognitive scores in the original observed data (red) versus the imputed datasets (blue). The overlapping curves confirm that the Multiple Imputation by Chained Equations (MICE) procedure preserved the original data structure and variance. [file DataSheet1.pdf]

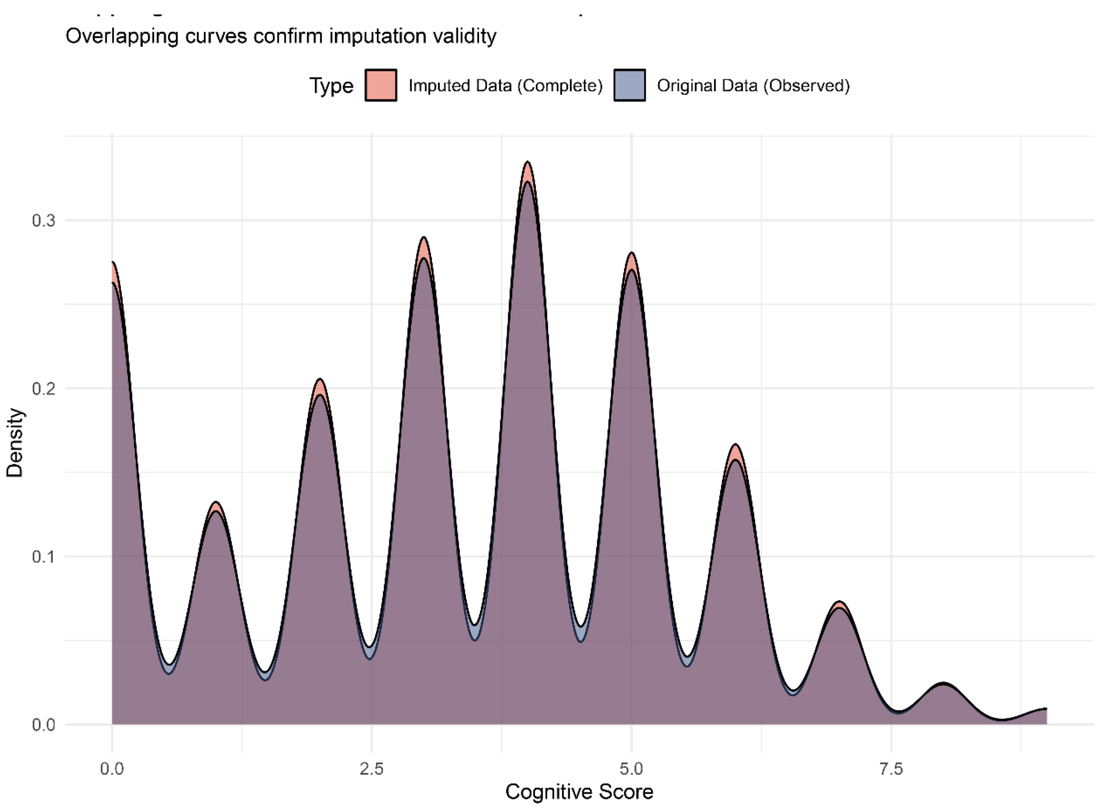

eFigure 1. Diagnostic Assessment of Multiple Imputation Validity.

Density plots comparing the distribution of cognitive scores in the original observed data (red) versus the imputed datasets (blue). The overlapping curves confirm that the Multiple Imputation by Chained Equations (MICE) procedure preserved the original data structure and variance.

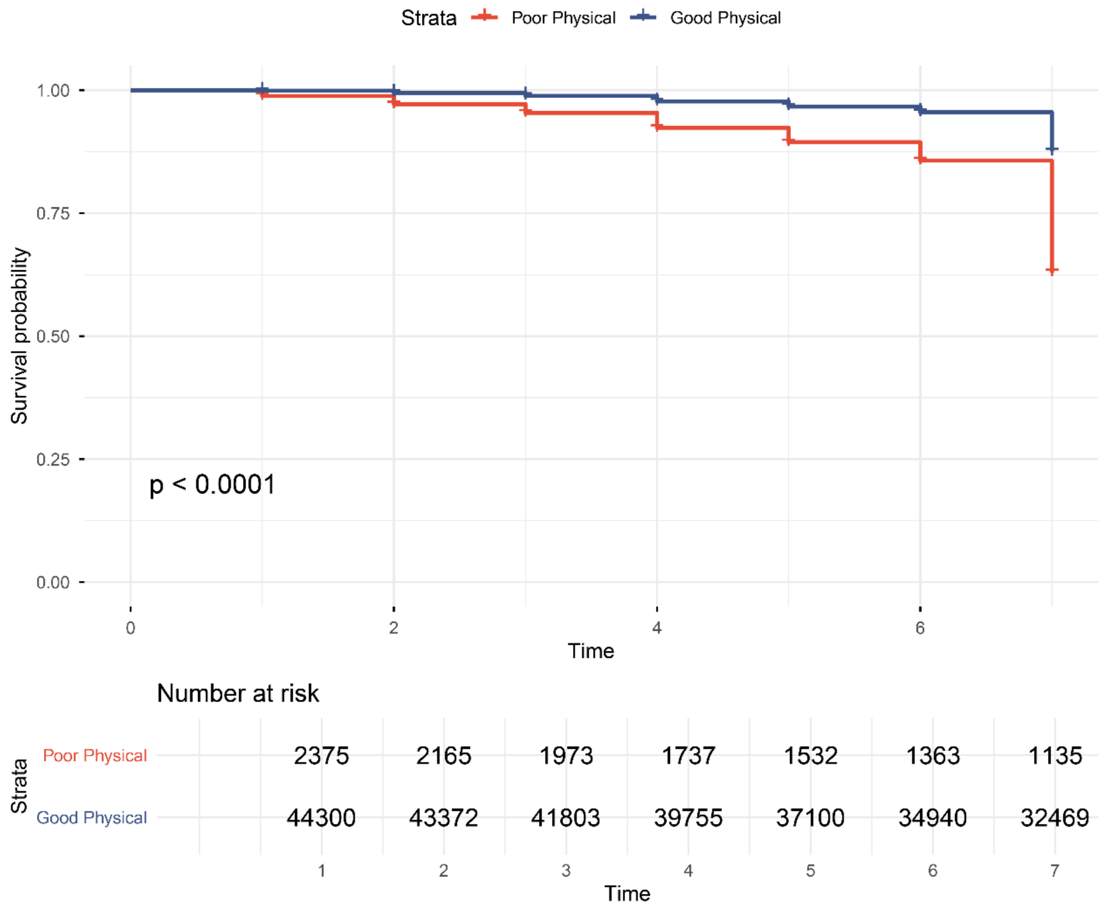

eFigure 2. Sensitivity Analysis: Survival Probability Excluding Participants with Stroke History.

Kaplan-Meier survival curves stratified by the four identified joint trajectory classes in a sub-cohort excluding all participants with a history of stroke ($N \approx 33{,}000$). The protective effect of good physical function remains highly significant ($p < 0.0001$), suggesting the association is not driven by cerebrovascular events.

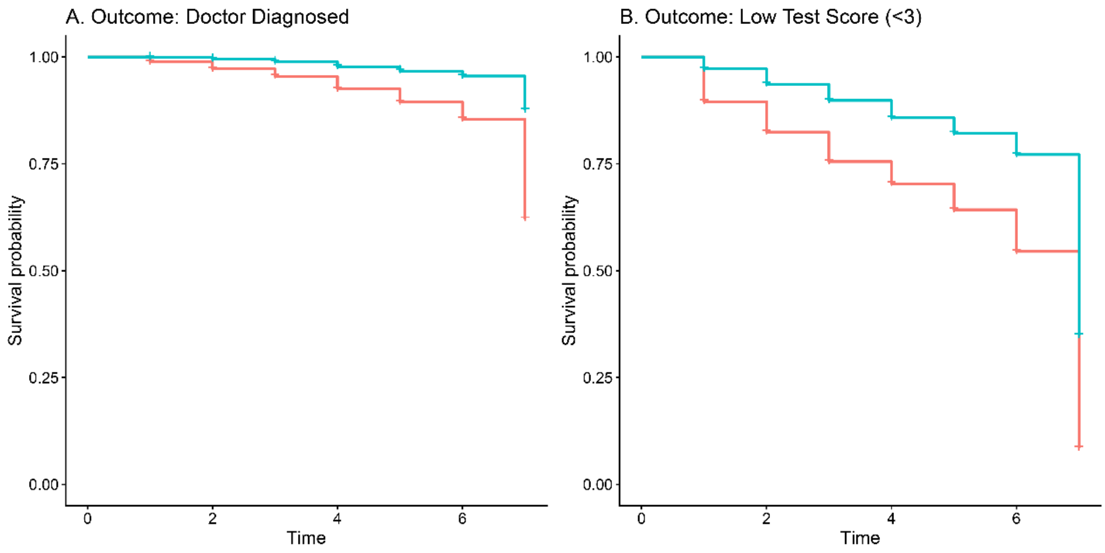

eFigure 3. Robustness of Findings Across Alternative Dementia Outcome Definitions.

Survival curves comparing associations when the outcome is defined as (A) Doctor-diagnosed dementia (Clinical definition) versus (B) Sensitivity analysis evaluating dementia risk using alternative physical frailty metrics. The pattern of risk stratification by physical function remains consistent across definitions.

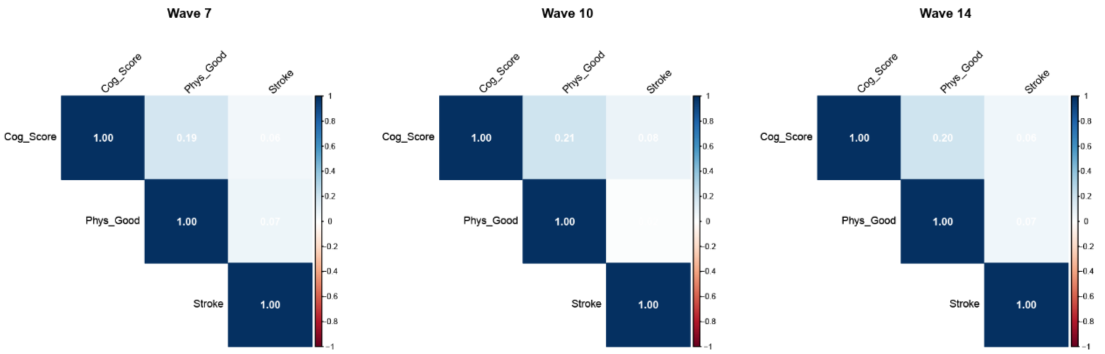

eFigure 4. Temporal Stability of the Physio-Cognitive Correlation Structure Across Waves.

Correlation heatmaps for key variables (Cognition, Physical Function, Comorbidities) at Wave 7, Wave 10, and Wave 14. The consistency of correlation coefficients (color

intensity) over the 8-year period indicates a stable phenotypic relationship rather than a transient association at end-of-life.

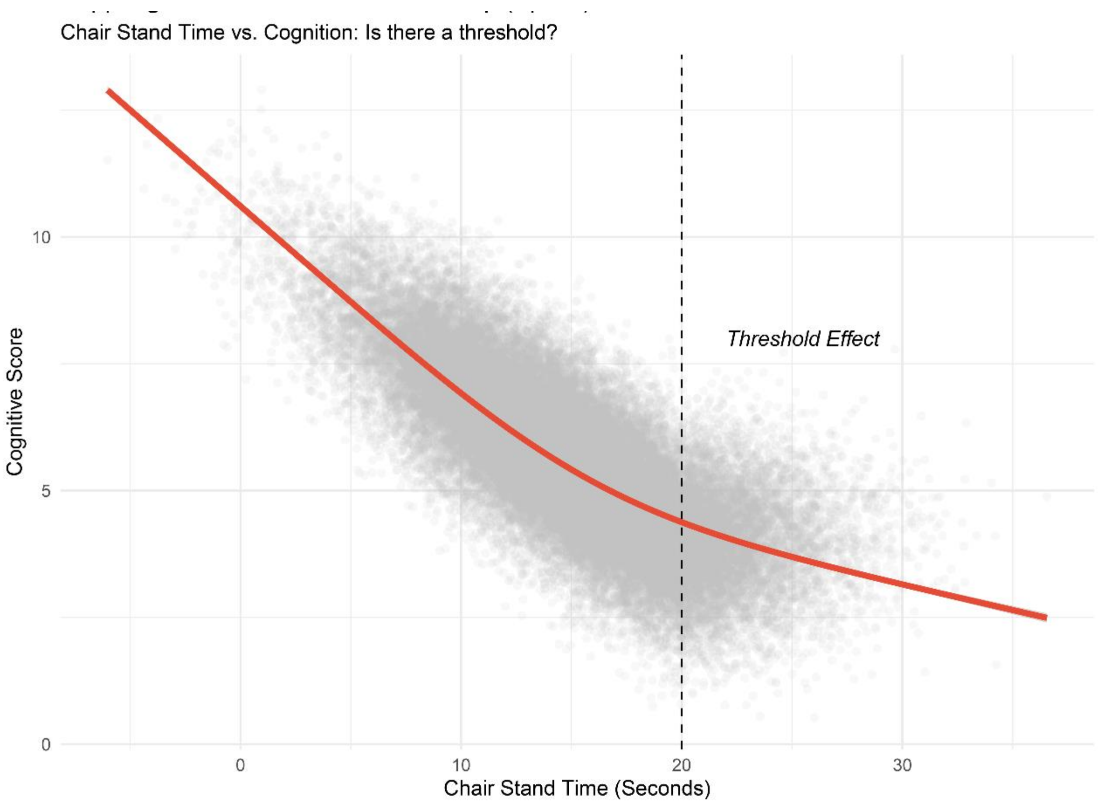

eFigure 5. Non-Linear Threshold Effects of Physical Performance on Cognitive Scores.

Generalized Additive Model (GAM) spline plot illustrating the relationship between Chair Stand time (seconds, continuous) and Cognitive Score. The shaded area represents the 95% confidence interval. A "threshold effect" is observed around 20 seconds (vertical dashed line), after which cognitive scores exhibit a precipitous decline, identifying a potential clinical cut-point.

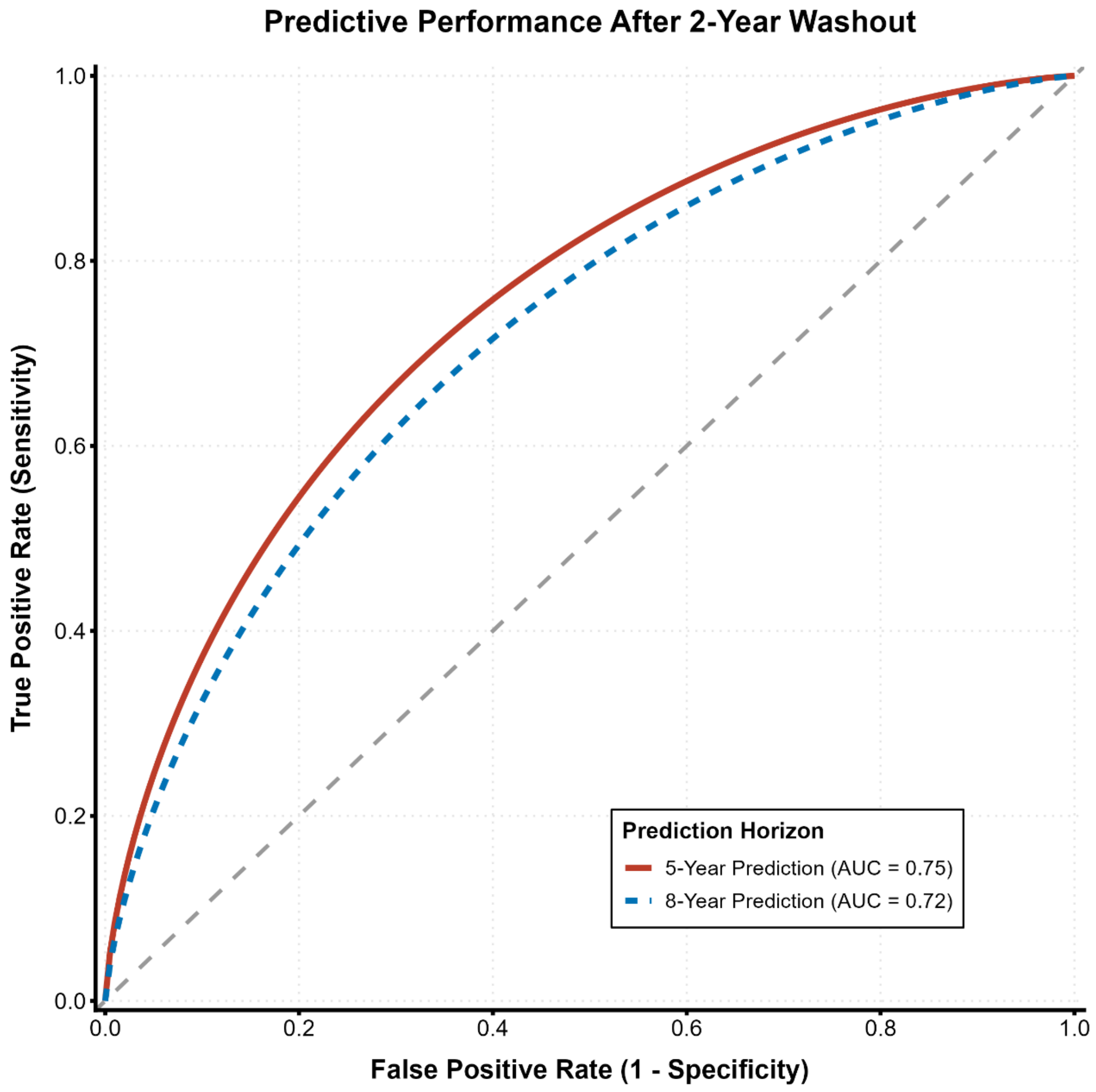

eFigure 6. Time-Dependent ROC Curves Following a 2-Year Washout Period.

Receiver operating characteristic (ROC) curves evaluating the discriminative ability of the full predictive model (incorporating Chair Stand Test trajectories, demographics, and clinical covariates) after excluding early-incident cases. Specifically, all participants who developed incident dementia within the first two years of follow-up were excluded to mitigate potential reverse causality and model overfitting. Despite the attenuation caused by removing these most easily detectable prodromal cases, the model maintains robust, long-term prospective accuracy,

achieving an Area Under the Curve (AUC) of **0.75 (95% CI: 0.71–0.79)** for 5-year prediction and **0.72 (95% CI: 0.68–0.76)** for the full 8-year horizon.
