# Supplementary material for: Joint trajectories of objective physical function and cognition and risk of incident dementia: a population-based cohort study
Source: Front Psychiatry. 2026 May 22;17:1804952. doi: 10.3389/fpsyt.2026.1804952 (PMC13236857; doi:10.3389/fpsyt.2026.1804952)
Supplement: Supplementary file 2 [file Table1.docx]

**Supplementary Appendix 1. Detailed Statistical Methods and Model Specification**

This appendix provides detailed technical specifications for the advanced statistical models utilized in this study, specifically addressing the modeling choices, estimation procedures, and our adherence to the Guidelines for Reporting on Latent Trajectory Studies (GRoLTS).

**1. Parallel-Process Latent Class Growth Analysis (LCGA) Specification**

To identify joint unobserved sub-populations (latent classes) with distinct longitudinal aging phenotypes, we conducted a parallel-process LCGA. This approach is superior to traditional two-step clustering as it jointly estimates trajectories across multiple domains (physical and cognitive) simultaneously, minimizing classification error.

**Software and Estimation:** The models were estimated using full-information maximum likelihood via the lcmm package (version 2.1.0) in R. Analytic survey weights were incorporated to account for the unequal probabilities of selection in the NHATS design.

**Link Functions:** To properly accommodate the different distributional properties of our outcome variables, distinct link functions were specified within the joint multlcmm framework. For the continuous/ordinal cognitive assessments (Delayed Word Recall and Clock Drawing Test), a **linear (Gaussian) link function** was utilized. For the binary objective physical function outcome (Chair Stand Test: Able vs. Unable), a **threshold (logit) link function** was employed.

**Polynomial Order (Trajectory Shape):** The start polynomial order for each domain’s trajectory was specified as a **linear functional form (time parameter of degree 1)**. During the model-building phase, we sequentially tested the inclusion of quadratic time parameters (degree 2). However, quadratic specifications either resulted in non-convergence issues or did not yield a clinically meaningful improvement in model fit indices (BIC/SABIC). Consequently, the linear specification was retained to favor model parsimony and avoid over-fitting sparse data at the tails of the follow-up period.

**Final Model Parameters:** The exact regression parameter estimates (intercepts and linear slopes) for each domain within the final 4-class model are detailed in **Supplementary eTable 2**.

**2. Adherence to GRoLTS Checklist (Model Selection Rationale)**

Our model selection, fitting, and reporting procedures were conducted strictly in accordance with the Guidelines for Reporting on Latent Trajectory Studies (GRoLTS). Below are the point-by-point specifications:

**Metric of Time:** The metric of time used for the trajectory analysis was the follow-up time in years from the baseline assessment (Round 7).

**Number of Latent Classes Evaluated:** The optimal number of classes was not prespecified. We systematically fitted and compared models ranging from 1 to 5 latent classes (see **Supplementary eTable 1**).

**Model Fit Evaluation:** Model selection was based on a comprehensive evaluation of established fit indices. We prioritized models with lower values of the Bayesian Information Criterion (BIC) and Sample-size Adjusted BIC (SABIC). The 4-class model exhibited the lowest BIC (71,563.8), indicating the optimal balance between model fit and parsimony.

**Classification Quality (Entropy and MPPs):** We acknowledge that Entropy is an index of the quality and certainty of class-assignment rather than strict class separation. To ensure high-quality assignment, we required an Entropy value >0.80. The final 4-class model yielded an Entropy of 0.83. Furthermore, the Mean Posterior Probabilities (MPPs) for all four classes ranged from 0.84 to 0.91, well above the acceptable threshold of 0.70, confirming robust and reliable classification for subsequent inferential analyses.

**3. Cross-Lagged Panel Model (CLPM) Specification**

To evaluate the temporal precedence between physical and cognitive decline without assuming a homogeneous population, we specified a Stratified CLPM.

**Software and Design Adjustment:** The model was constructed using the lavaan package and subsequently adjusted for the complex survey design (strata, primary sampling units, and analytic weights) using the lavaan.survey package.

**Path Specification:** The model estimated autoregressive paths (e.g., physical function at wave *t* predicting physical function at wave *t+1*) and cross-lagged paths (e.g., physical function at wave *t* predicting cognitive function at wave *t+1*, and vice versa). The model was stratified by the latent classes identified in the LCGA to reveal heterogeneous temporal dynamics across different risk phenotypes.

**4. Survey-Weighted Survival Mediation Analysis**

To robustly test the "Life Space Constriction" hypothesis with incident dementia hazard as the strict endpoint, we conducted a counterfactual-based survival mediation analysis.

**Pathways:** The natural direct effect (NDE) and natural indirect effect (NIE) were estimated on the log-hazard scale. The exposure was baseline physical function (impaired vs. good), the mediator was the continuous life space score at follow-up, and the outcome was time-to-incident dementia.

**Variance Estimation:** Because the integration of standard complex survey weighting into non-linear mediation is statistically challenging, we utilized a custom bias-corrected bootstrapping procedure with 1,000 iterations. Critically, to preserve the complex survey design, resampling was conducted at the Primary Sampling Unit (PSU) level within each design stratum, ensuring valid standard errors and confidence intervals for the indirect effects. Results across all latent subgroups are reported in **Supplementary eTable 4**.
