# Supplementary material for: Joint trajectories of objective physical function and cognition and risk of incident dementia: a population-based cohort study
Source: Front Psychiatry. 2026 May 22;17:1804952. doi: 10.3389/fpsyt.2026.1804952 (PMC13236857; doi:10.3389/fpsyt.2026.1804952)
Supplement: Supplementary file 3 [file Table2.docx]

**eTable 1. Comparison of Baseline Characteristics Between Included and Excluded Participants**

| Baseline Characteristics | Final Included Sample (N=6,175) | Excluded Sample (N=1,480) | P-value | SMD |
| --- | --- | --- | --- | --- |
| Age, years (mean ± SD) | 75.2 ± 6.8 | 81.4 ± 8.2 | < 0.001 | 0.824 |
| Gender (Female), No. (%) | 3,457 (56.0%) | 962 (65.0%) | < 0.001 | 0.185 |
| Education, No. (%) |  |  | < 0.001 | 0.245 |
| ≤ High School | 2,161 (35.0%) | 784 (53.0%) |  |  |
| > High School | 4,014 (65.0%) | 696 (47.0%) |  |  |
| Race/Ethnicity, No. (%) |  |  | 0.012 | 0.134 |
| Non-Hispanic White | 4,968 (80.5%) | 1,125 (76.0%) |  |  |
| Non-Hispanic Black | 514 (8.3%) | 178 (12.0%) |  |  |
| Other | 693 (11.2%) | 177 (12.0%) |  |  |
| Comorbidities, No. (%) |  |  |  |  |
| Hypertension | 3,148 (51.0%) | 858 (58.0%) | < 0.001 | 0.141 |
| Diabetes | 1,715 (27.8%) | 518 (35.0%) | < 0.001 | 0.155 |
| Heart Disease | 2,023 (32.8%) | 592 (40.0%) | < 0.001 | 0.150 |
| Stroke | 597 (9.7%) | 296 (20.0%) | < 0.001 | 0.298 |

**Footnotes:** An SMD > 0.1 indicates a meaningful imbalance between the two groups. P-values were calculated using Student's t-test for continuous variables and Chi-square tests for categorical variables. The excluded sample (N = 1,480) primarily consists of individuals who met the criteria for prevalent dementia at baseline, lacked key covariates, or were completely lost to follow-up.

**eTable 2. Model Fit Indices for Parallel-Process Latent Class Growth Analysis of Physical and Cognitive Trajectories**

| Number of Classes (k) | Parameters | Log-likelihood | AIC | BIC | SABIC | Entropy | Class Prevalence (%) | Mean Posterior Probability (MPP) |
| --- | --- | --- | --- | --- | --- | --- | --- | --- |
| 1 Class | 8 | -39,520.4 | 79,056.8 | 79,111.6 | 79,086.2 | N/A | 100% | N/A |
| 2 Classes | 13 | -37,145.2 | 74,316.4 | 74,405.5 | 74,364.1 | 0.74 | 72.5 / 27.5 | 0.81 / 0.85 |
| 3 Classes | 18 | -36,210.8 | 72,457.6 | 72,580.9 | 72,523.8 | 0.78 | 60.1 / 25.4 / 14.5 | 0.84 / 0.79 / 0.88 |
| 4 Classes (Selected) | 23 | -35,680.1 | 71,406.2 | 71,563.8 | 71,490.7 | 0.83 | 55.2 / 19.8 / 15.1 / 9.9 | 0.89 / 0.84 / 0.86 / 0.91 |
| 5 Classes | 28 | -35,665.5 | 71,387.0 | 71,578.9 | 71,489.9 | 0.76 | 45.3 / 20.1 / 14.8 / 11.2 / 8.6 | 0.78 / 0.75 / 0.81 / 0.80 / 0.71 |

**Footnote:** The parallel-process Latent Class Growth Analysis (LCGA) was estimated using full-information maximum likelihood via the *lcmm* package. To answer the reviewer's specific queries: The model utilized a linear link function (Gaussian) for the continuous cognitive scores and a threshold/logit link for the categorical physical function outcomes. The start polynomial order for each trajectory was specified as a linear functional form (time parameter of degree 1), as quadratic terms did not significantly improve model fit. The 4-class model was selected as the optimal solution because it demonstrated the lowest BIC value (71,563.8), high Entropy (0.83) indicating high quality of class-assignment, and high Mean Posterior Probabilities (MPPs) for all trajectory classes (ranging from 0.84 to 0.91).

**eTable 3. Final Model Regression Parameters for the 4-Class Parallel-Process Latent Class Growth Analysis**

| Trajectory Class | Domain | Intercept Estimate (SE) | P-value | Linear Slope Estimate (SE) | P-value |
| --- | --- | --- | --- | --- | --- |
| Class 1: Resilient Agers | Physical Function | 2.85 (0.12) | <0.001 | -0.02 (0.01) | 0.125 |
|  | Cognitive Function | 7.95 (0.24) | <0.001 | -0.05 (0.02) | 0.068 |
| Class 2: Physical Decliners | Physical Function | 2.45 (0.15) | <0.001 | -0.45 (0.04) | <0.001 |
|  | Cognitive Function | 7.60 (0.28) | <0.001 | -0.10 (0.03) | 0.012 |
| Class 3: Cognitive Decliners | Physical Function | 2.70 (0.14) | <0.001 | -0.08 (0.02) | 0.045 |
|  | Cognitive Function | 6.85 (0.26) | <0.001 | -0.65 (0.06) | <0.001 |
| Class 4: Dual Rapid Decliners | Physical Function | 1.85 (0.18) | <0.001 | -0.68 (0.05) | <0.001 |
|  | Cognitive Function | 5.20 (0.32) | <0.001 | -0.95 (0.08) | <0.001 |

**Footnote:** Standard errors (SE) are derived from the maximum likelihood estimation. The intercept represents the baseline marginal mean score for each domain within the specified latent class, and the linear slope represents the annual rate of change over the 8-year follow-up period.

**eTable 4. Full Multivariable Survey-Weighted Cox Proportional Hazards Model for Incident Dementia**

| Predictor | Hazard Ratio (HR) | 95% Confidence Interval | P-value |
| --- | --- | --- | --- |
| Latent Trajectory Class |  |  |  |
| Class 1: Resilient Agers | 1.00 (Reference) | — | — |
| Class 2: Physical Decliners | 1.85 | 1.45 – 2.35 | <0.001 |
| Class 3: Cognitive Decliners | 2.45 | 1.95 – 3.10 | <0.001 |
| Class 4: Dual Rapid Decliners | 4.85 | 3.90 – 6.05 | <0.001 |
| Sociodemographics |  |  |  |
| Age (≥ 75 vs. <75 years) | 1.62 | 1.48 – 1.78 | <0.001 |
| Gender (Female vs. Male) | 1.28 | 1.20 – 1.35 | <0.001 |
| Race/Ethnicity (Black vs. White) | 1.31 | 1.23 – 1.40 | <0.001 |
| Race/Ethnicity (Other vs. White) | 0.93 | 0.86 – 1.01 | 0.093 |
| Baseline Clinical Comorbidities |  |  |  |
| Stroke History | 1.45 | 1.25 – 1.68 | <0.001 |
| Hypertension | 1.15 | 1.06 – 1.25 | 0.012 |
| Diabetes Mellitus | 1.22 | 1.12 – 1.34 | <0.001 |

**Footnote:** Model rigorously accounts for the complex survey design (strata, primary sampling units, and analytic weights). HRs represent the risk of incident dementia relative to the reference group, adjusting for all demographic and clinical variables listed in the table. Sociodemographic and Stroke HRs are anchored to the baseline model parameters.

**eTable 5. Survey-Weighted Survival Mediation Analysis of Life Space Constriction Across All Latent Trajectory Subgroups**

| Trajectory Subgroup | Total Effect on Incident Dementia (Log-Hazard) | Direct Effect | Indirect Effect (via Life Space) | Proportion Mediated (%) | Indirect Effect P-value |
| --- | --- | --- | --- | --- | --- |
| Overall Cohort | -1.25 | -1.04 | -0.21 | 16.8% | <0.001 |
| Class 1: Resilient Agers | -0.18 | -0.15 | -0.03 | 16.6% | 0.345 |
| Class 2: Physical Decliners | -0.85 | -0.68 | -0.17 | 20.0% | 0.012 |
| Class 3: Cognitive Decliners | -0.55 | -0.48 | -0.07 | 12.7% | 0.085 |
| Class 4: Dual Rapid Decliners | -1.88 | -1.45 | -0.43 | 22.8% | <0.001 |

**Footnote:** Mediation effects were estimated using bias-corrected bootstrapping (1,000 iterations) incorporating the NHATS complex survey design. Exposure: Baseline Physical Function; Mediator: Follow-up Life Space; Outcome: Time-to-Incident Dementia. Consistent with clinical expectations, the mediation pathway via life space constriction was highly significant in overall cohort and physically declining phenotypes (Classes 2 and 4) but weak and non-significant in the Resilient Agers class.

**eTable 6. Linear Mixed Model Evaluating the "Double Hit" Interaction Between Physical Frailty and Sensory Impairment**

| Fixed Effects on Rate of Cognitive Decline (Slope) | Estimate (β) | Standard Error (SE) | P-value |
| --- | --- | --- | --- |
| Main Effect: Poor Physical Function | -0.42 | 0.05 | <0.001 |
| Main Effect: Vision Impairment | -0.25 | 0.08 | 0.003 |
| Main Effect: Hearing Impairment | -0.16 | 0.07 | 0.024 |
| Interaction: Physical Function × Vision Impairment | -0.15 | 0.07 | 0.040 |
| Interaction: Physical Function × Hearing Impairment | -0.08 | 0.06 | 0.215 |

**Footnote:** Model adjusted for baseline age, gender, race, and systemic comorbidities. The significant interaction term (P = 0.04) indicates that the steepness of cognitive decline associated with poor physical function is significantly exacerbated when concurrent vision impairment is present, supporting the "Double Hit" hypothesis.

**eTable 7. Sensitivity Analysis of Dementia Risk by Frailty Definition**

| Characteristic | Definition 1: Strict (Chair Stand Only) HR (95% CI) | P-value | Definition 2: Broad (Inclusive Criteria) HR (95% CI) | P-value |
| --- | --- | --- | --- | --- |
| Good Physical Function | 0.29 (0.26 – 0.31) | <0.001 | 0.30 (0.27 – 0.33) | <0.001 |

**Footnote:** Cox proportional hazards models adjusted for age and gender. Definition 1 (Strict) classifies physical function solely based on the objective chair stand test (Able vs. Unable). Definition 2 (Broad) incorporates additional subjective fatigue or broader frailty criteria. The protective effect of good physical function remains robust across definitions. Abbreviations: HR, Hazard Ratio; CI, Confidence Interval.
